# Transdisciplinary Communities of Practice to Resolve Health Problems in Southeast Asian Artisanal and Small-Scale Gold Mining Communities

**DOI:** 10.3390/ijerph19095422

**Published:** 2022-04-29

**Authors:** Win Thiri Kyaw, Masayuki Sakakibara

**Affiliations:** 1 Research Institute for Humanity and Nature, Kyoto 603-8047, Japan; sakaki@chikyu.ac.jp; 2 Graduate School of Science and Engineering, Ehime University, Matsuyama 790-8577, Japan

**Keywords:** artisanal and small-scale gold mining, ASGM, TDCoPs, transdisciplinary, mercury, Southeast Asia

## Abstract

Artisanal and small-scale gold mining (ASGM) has been a major part of people’s livelihood in the rural areas of many developing countries, including those in Southeast Asia (SEA). Nevertheless, because of the use of mercury, ASGM activities have significant local and global adverse impacts on the environment and ASGM community health. Although there have been many monodisciplinary projects by academic researchers and governments to solve the environmental and health problems in SEA ASGM communities, they have not been sufficient to solve the complex socioeconomic problems. This review first outlines the nature of the SEA ASGM activities and the consequent environmental, community health, and socioeconomic problems and then introduces an approach using transdisciplinary communities of practice that involves both academic and nonacademic participants to relieve these wicked ASGM problems and to improve the environmental governance and community health in ASGM communities in SEA.

## 1. Introduction

Artisanal and small-scale gold mining (ASGM) is an informal occupational sector in many rural parts of developing nations. It relies on unskilled labor to mine and process gold, particularly in areas where agricultural income alone is unable to support community livelihoods. The International Labor Organization (ILO) reported that ASGM activities are labor-intensive, involve a small number of people, and rely on basic equipment. Globally, over 100 million people are directly or indirectly involved in ASGM for their livelihoods. Although ASGM contributes to poverty alleviation and generates national income, it has also been negatively associated with social, environmental, and health issues [1,2]. On a social level, catastrophic devastation, gender inequality, and child labor issues occur as a result of the discovery of deposits and the consequent influx of small-scale miners into formerly native or rural communities [2]. Nevertheless, the social issues differ between continents; for example, the primary challenges in Africa are AIDS and sustainable community development; in Asia/Pacific, the challenges are related to intercultural elements and cultural rights; and in Latin American/Caribbean, the most important issues are the environment, indigenous peoples, and legal aspects [2].

Many developing countries in Southeast Asia (SEA) practice ASGM because of poverty and tradition. Most ASGM is practiced in Indonesia, the Philippines, and Myanmar, with a significantly smaller percentage of people working in ASGM in Thailand, Cambodia, and Laos. The more specific ASGM profiles in these countries are discussed later in this paper.

Similar to other countries involved in ASGM activities, mercury (Hg) is commonly applied as a cheap, rapid method for extracting gold, although it is highly toxic and can have serious health consequences for miners, their families, and the neighboring communities. ASGM sectors in over 70 countries have been found to have the largest global Hg emissions at 640 to 1350 tons annually, and a recent United Nations Environment Program (UNEP) global mercury assessment found that global ASGM activities were increasing [3]. Although Hg is illegal in some countries, as it is affordable and easy to use, it continues to be widely used. The Hg used in SEA is directly sourced from Spain and China or from less transparent transit routes through Singapore [4]. In Indonesia, approximately 280 tons of illegal Hg was imported in 2010 [5]; however, UN Comtrade (2020), an international UN branch that reports goods that were officially exported and imported by countries [6], reported that Indonesia had shifted from importing to exporting Hg in 2015 because of its development of national cinnabar (HgS) mines [7]. A previous report in 2002 on the estimation of annual Hg emissions from ASGM activities found that Indonesia emitted the most of all SEA countries at 145 tons, followed by the Philippines with 25.0 tons, Cambodia and Vietnam with 7.5 tons each, Myanmar with 6.5 tons, Malaysia with 3.5 tons, Thailand with 1.5 tons, and Laos with 1.3 tons [8].

While Hg is useful for extracting gold, it causes significant environmental pollution and is a health hazard for the miners and the community. Hg (elemental Hg) is used to bind the gold in the ore, which is then known as a gold–Hg amalgam. The amalgam is then smelted to extract the gold [9], which emits an Hg vapor into the atmosphere, where it can be oxidized into ionic Hg. It can either fall into water bodies directly or be deposited on the land’s surface and washed into an aquatic system by run off. When ionic mercury enters water, it undergoes three main transformations. First, it may be volatilized back into elemental Hg. The second channel involves adsorption into sediments in lakes, rivers, and reservoirs. Finally, in anaerobic conditions at the bottom of the water bodies and the sediment–water interface, the sulfate-reducing bacteria may methylate ionic Hg to become methylmercury (MeHg), the most toxic form of Hg, which can be ingested by planktons, after which it enters the food chain through bioaccumulation in long-lived predatory species such as sharks and shellfish [10]. Thus, fish can collect significant levels of MeHg, which can be consumed by humans or wildlife. MeHg is found in fish protein and is not degraded by cooking or washing [11]. Aquatic changes in Hg occur in both directions. In certain cases, these processes can be reversed; thus, several types of Hg can be found in aquatic systems. However, increased Hg contents were discovered not only in marine food but also in rice samples of ASGM areas in Indonesia [12,13], which may occur through the absorption of MeHg by the paddy roots, and where it is stored in the rice grain until it reaches ripening [14]. This may also occur in other agricultural lands due to the waste discharged from the process of ASGM.

In addition, Hg vapor can be inhaled by miners during the smelting process, which is their main Hg exposure route [15]. Thus, regardless of whether they are directly engaging in ASGM activities, ASGM community health is doubly affected by the direct inhalation of Hg vapor and by the consumption of contaminated fish and shellfish. Children are more susceptible to the adverse health impacts of Hg, and even before they are born, they can suffer neurological disorders from prenatal Hg dietary exposure from the consumption of marine fish by their mothers.

Despite these serious issues, continuing poverty in SEA means that ASGM activities are expected to increase, which will escalate these “wicked problems”; therefore, a sound, sustainable solution is needed. It is hypothesized that the major contributors to these wicked problems are (1) the lack or insufficient awareness in SEA ASGM communities because of poor information and (2) ineffective approaches by academic researchers, local experts, governments, and other stakeholders in tackling these problems. Thus, solutions to these wicked ASGM problems in SEA are needed in order to address the ASGM contributing factors, the causative ASGM hazards, and the health hazards in mining communities.

## 2. Methods

To identify relevant studies, we conducted searches related to information on the ASGM of SEA countries and their socioeconomic and community health hazards in search engines such as PubMed, Google Scholar and Scopus. Terms such as “mercury”, “ASGM”, “socio-economic”, “health”, “health impact”, “health assessment”, and “mercury intoxication” were included in the search. The reports were then filtered by the name of SEA countries, reports with the clinical findings, and the usage of English. The reports with clinical assessments were considered relevant for the summary of health assessments.

## 3. ASGM Profiles in SEA Countries

### 3.1. General Overview of Hg Usage in ASGM

Although Hg has serious environmental and health impacts, it is widely used as part of the ASGM process because it is cheap, easily available, and easy to handle. Developing countries such as those in SEA use Hg in both whole-ore-amalgamation and concentration methods. The whole-ore-amalgamation method results in higher Hg emissions from the tailings than the concentration method [16]. With the whole-ore-amalgamation method, the Hg come into contact with 100% of the ore, with four parts of Hg being used for every one part of gold; that is, the gold-to-Hg ratio is 4:1, and it can be even higher, such as 20:1 or 50:1 [16]. However, with the concentration method, the gold is initially reduced to a smaller quantity before the amalgamation is performed, which is commonly done using gravity. Then, only the concentrations that have the heaviest minerals and gold are treated with the Hg, which means that the Hg-to-gold ratio is substantially lower than in the whole ore amalgamation method (usually 1:1 vs. 1.3:1), and significantly smaller quantities of Hg remain in the tailings [16]. Thus, the nature and the degree of environmental destruction and damage to human health depend on the type of minerals in the local areas and the type of ASGM processes being used.

### 3.2. Indonesia

ASGM activity in Indonesia is illegal, and Indonesian health and environmental regulations have banned ASGM. Nonetheless, Indonesia is well known for having the most ASGM activities in SEA, with more than 850 mining sites and 200,000 miners [12,17,18]. ASGM involves panning, dredging, or using high-pressure pumps on river banks, open pits, or vertical excavations to expose the secondary or tertiary alluvial ores or to physically extract the ore from the neighboring hills or mountains using traditional instruments, such as wide hoes, bars, and basic pulleys. Hammers and other similar tools are used to hand-smash the ore, and homemade mechanical crushers are used in some processing industries. The ore is then physically loaded into sacks and hauled to the processing facilities, where the crushed ore (30–40 kg) is put into a trommel, a steel mill grinder, where the gold is extracted from the ore using water and decimeter-sized boulders and milled for 3–4 h until the material is fine enough to release the gold. Then, roughly 500 g of Hg is fed into the rotating trommel for approximately 30 min to complete the amalgamation [19], after which the material is discharged, separated, and washed with bare hands to separate the gold from the nonbinding Hg while keeping the sediment aside for a second use. The resulting amalgam is burned inside or outside dwellings and gold shops, which emits Hg vapor into the surrounding area. Approximately 20–30% of gold is obtained from the amalgamation method.

In Indonesia, similar to many other nations in Latin America and Africa, the processing facilities in Indonesia offer free or low-cost ore amalgamation to miners in exchange for the tailings [20]. The processing facility keeps the Hg-contaminated tailings to recover the residual gold using cyanide (CN), which is significantly more effective than Hg at extracting fine gold particles. Usually, 80–90% of the gold in the tailings is retrieved in this way. Nevertheless, CN dissolves the remaining Hg in the tailings, which generates the mercury cyanide complexes, Hg(CN)_(2+N)_^(-N)^ (aq) compound, and causes significantly greater aquatic biota pollution than the elemental Hg [21]. In rivers, the sediment is washed and panned by women and children, and the gold is extracted from the sediment using Hg [19]. In some areas, the muddy water that is tainted by Hg-contaminated tailings is released directly into the local waterways, which then drains into the rice paddy fields and fish ponds [12]. Thus, Hg contaminates the soil, the sediment, the water and biota, the plants, the tree bark, and even cattle [20,22,23,24,25]. As gold mining activities involve materials extraction, other heavy metals are also exposed, such as arsenic (As), cadmium (Cd), lead (Pb), cobalt (Co), manganese (Mn), and zinc (Zn) [26,27]. High levels of As and Pb that exceed the safe drinking water limit defined by the WHO have been detected in water samples from the Bone River, which is close to an ASGM site [28].

Soil remediation solutions for Hg-polluted soils have been developed, such as phytoremediation using green plants and their microbiome, to remediate polluted areas [29]. However, the expense of traditional phytoremediation increases each year, and the owners of polluted lands lose money throughout the cleanup process, limiting its practical implementation. Therefore, recently, high-biomass crops such as Indian mustard, maize, sunflower, and sweet sorghum have been grown to be able to remediate heavy metals for practical and economic reasons [30]. A field study in Indonesia suggested that KCS105 sweet sorghum might be a promising energy crop for phytoremediation of mercury-polluted soil because it grew well on mercury-contaminated soil and accumulated mercury in its root and shoot. Furthermore, Agrobacterium tumefaciens inoculation increased the phytoremediation efficacy of Hg to 934 mg/ha [31].

### 3.3. The Philippines

The Philippines ranks 20th in global gold production, with 70% of this coming directly from the estimated 500,000 ASGM sites operating in over 40 of its 81 provinces. The ILO estimated that around 19,000 children work in ASGM in the Philippines [32]; however, after the Philippines committed to legalizing ASGM activities, several measures were introduced between 2016 and 2020 to end ASGM child labor, such as the CARING Gold Mining Project in the Philippines operated by the ILO and executed with support from BanToxics and the United States Department of Labor [33]. Gold ore is mined mostly around Diwalwal in Mindanao, which is one of the Philippines’ major islands. Dominated by Mount Diwata, Diwalwal is a prominent gold town of 15,000 inhabitants, in which gold mining has been conducted for over a century [25]. The Mount Diwata ASGM area comprises small industrial complexes, such as ball mill and cyanidation facilities, as well as stores and housing, all of which are scattered around the site, with their wastes, including human waste, being thrown into the rivers or discarded. Small communities in the Diwalwal community living area crush the mining ore into powder, and Hg is added to make the gold–Hg amalgam, which is later burned by small local companies or in the miners’ homes. Thus, Hg tailings are found throughout the region [34]. Tuberculosis is the leading cause of death in Diwalwal, and the local health clinic, which has had only midwives and “helots” and no doctor for years, is therefore underequipped and unable to diagnose or treat Hg toxicity.

### 3.4. Myanmar

ASGM is mainly practiced in 300 officially recorded areas in Kachin State, Sagaing Region, Mandalay Region, and Bago Region, in which an estimated 730,000 people work [35]. The main ASGM activities are panning, river mining with bucket dredges, suction dredging, hydraulic mining, open-pit mining for alluvial and colluvial gold deposits [36], and underground mining for hard rock deposits [37,38,39]. CN is often used in underground mining to crush rocks and dissolve gold [35]. The final gold is recovered using Hg, which is either collected for a second use or discharged into the waterways. Similar to the ASGM activities in Indonesia, to retrieve the gold, the Hg amalgam is burned in local gold shops, inside the houses, or in the open air [37]. The Hg emitted from the ASGM activities has polluted the environment [36,37] and has contaminated groundwater and the atmosphere [39]. High concentrations of heavy metals, such as As, Cd, Pb, and Hg, have also been found in ASGM site soil samples [40].

### 3.5. Other SEA Countries

Vietnam has approximately 63,000 ASGM workers, Cambodia has approximately 6000, and Laos has approximately 3000 [8]. The ASGM activities in the Lao PDR are relatively unknown; however, the Department of Gold Mining has claimed that there are ASM sites in Borikhamxay, Saravanh, Vientiane, and Luang Prabang provinces, although the extent and usage of Hg are unclear. A baseline study by Earth Systems Lao in Luan Prabang Province, Lao PDR, found that the ASGM activities were primarily conducted by families, with the ore/alluvium extraction normally performed by men using shovels and chisels, with the women and children carrying the ore to bowls and sluice boards, panning the ore, and extracting the gold at home by applying the Hg at the panning stage [41]. Similarly, ASGM in Cambodia is practiced at the family level, with most gold extraction activities occurring in the northeast part of the country. The estimated annual usage of Hg is from 34.5 to 1182 kg [42], and CN is also used [43].

## 4. Socioeconomic Hazards and Community Health Hazards Due to ASGM Activities

### 4.1. Socioeconomic Hazards Due to ASGM Activities

Due to the lack of infrastructure, mining communities have many socioeconomic problems, such as (1) conflicts between the native miners and the migrant miners; (2) gender inequality, which means that females have less income and less opportunity; and (3) poor or no educational support for children or the adults [44].

### 4.2. Health Hazards from Exposure to Hg and Other Heavy Metals

As discussed above, ASGM workers are exposed to elemental Hg mainly by inhaling the Hg vapor during the amalgamation and amalgam-burning processes. There are two ways in which elemental Hg in the atmosphere can be changed into two other forms of Hg: oxidizing into inorganic mercury salts (Hg^+^ and Hg2^+^) and methylating into methylmercury (MeHg), which has been found in fish [34]. The burning of the amalgam in homes or in open air exposes ASGM workers, their families, and their neighbors to the Hg vapor, which passes through the alveolar membrane, is absorbed into the blood, and travels to tissues, primarily affecting the respiratory system [45]. The major elemental Hg absorption takes place in the lungs (80%), after which it rapidly travels into the blood and other organs [46]. The inhaled Hg vapor can also cross the blood–brain barrier and blood–placenta barrier and accumulates in the central nervous system. Elemental Hg is primarily deposited in the brain and kidneys in its oxidized form, with the kidney being the organ that can be most damaged from repeated exposure [46].

Thus, ASGM communities can be exposed to Hg and other heavy metals, such as Pb, Cd, Co, and Mn. The International Agency for Research on Cancer has classified As as a Category 1 carcinogen that can cause bladder, skin, and lung cancers. Previous ecological research has linked high As concentrations in the soil to an increased risk of cancer. Lead is also a carcinogen that can cause kidney damage, hypertension, and a decrease in mental abilities. Furthermore, chronic low-level Mn exposure has been linked to an increase in Parkinsonism in exposed populations, and cobalt exposure can cause lung cancer, cardiomyopathy, and hearing and vision loss [47].

#### 4.2.1. Acute Effects from Exposure to Elemental Hg

Dermatitis can result from acute Hg exposure. After inhaling elemental Hg vapors, people may suffer from coughs, chills, fevers, shortness of breath, and gastrointestinal symptoms such as nausea, vomiting, and diarrhea, which can be followed by a metallic taste, dysphagia, salivation, weakness, headaches, and visual disorders [48]. Lungs can also be severely damaged from acute high-level exposure, and in severe cases, the resulting hypoxia can result in death. Reportedly, two children died, and their parents suffered from severe respiratory distress because of gold processing in a kitchen with poor ventilation [49].

#### 4.2.2. Chronic Effects from Exposure to Elemental Hg through Inhalation and MeHg through the Food Chain

When elemental Hg accumulates in the central nervous and renal systems, people are mainly affected by chronic intoxication, the major clinical presentations for which are unintentional or intentional tremors, psychological disturbances or erethism, proteinuria, and gingivitis [50]. Erethism can cause behavioral changes, such as irritability, low self-confidence, depression, apathy and shyness, and proteinuria resulting in tubular damage [46]. Other disorders include allergies or autoimmunity because of the reduced resistance to infection and cancers [51]. Children are particularly sensitive to exposure from eating MeHg-contaminated seafood. During pregnancy, MeHg bioaccumulates in fish, causing neurodevelopmental issues in the unborn child. The fetal brain is particularly vulnerable to transplacental exposure. Mental retardation, seizures, visual and hearing loss, developmental delays, language difficulties, and memory loss are all neurological signs. Chronic Hg exposure in children causes acrodynia, a condition marked by red and aching limbs [52,53].

### 4.3. Other Health Hazards

Most studies have focused on Hg-related health impacts in ASGM miners and communities; however, there are also other community health hazards [54], such as respiratory damage from the silica dust from drilling the ores (silicosis) [55], which increases the risk of tuberculosis from silicosis, and malaria, noise exposure, and injury, all of which are aggravated by the lack of infrastructure and the crowded living conditions.

### 4.4. ASGM Health Assessments in SEA Countries

There is no universal diagnosis for chronic Hg intoxication. Many of the health surveys in ASGM communities in Indonesia, the Philippines, and the Laos PDR have been conducted with the support of the United Nations Industrial Development Organization (UNIDO) Global Mercury Project. Clinical examinations and biomonitoring of Hg levels in the hair, blood, and urine have found instances of chronic elemental Hg intoxication and chronic MeHg intoxication from the food chain that has caused the loss of peripheral vision; ataxia; pins and needles in the hands, feet, and around the mouth; speech and hearing impairments; and muscle weakness. Because they are easily accessible, hair Hg levels are commonly analyzed to assess the Hg exposure in ASGM miners and communities; however, hair and blood Hg levels generally indicate the presence of MeHg through contamination of the food chain, and urine Hg levels indicate the occupational elemental Hg exposure from the ASGM activities.

Table 1 summarizes the findings of surveys conducted in SEA countries, which include clinical chronic Hg intoxication analyses in miners and ASGM communities and, as suggested by the German Human Biomonitoring Commission, the collection of biomonitoring samples that assessed the Hg content in hair (µg/g). Normal levels are below 1.0 µg/g, alert levels are 1.0–5.0 µg/g, and levels over 5.0 µg/g indicate a substantial health risk [56]. Most studies have found that the miner and community participants were suffering from Hg health impacts.

Overall, however, there have been few ASGM health impact evaluation projects in SEA. To the best of our knowledge, there was only one preliminary health survey conducted in Myanmar in 2020, which included clinical examinations and hair sample analyses [57] and only one online health survey [58] of the ASGM communities in the Mandalay Region, Thabeikkyin Township, Chaung Gyi Village. However, no clinical health assessments have been conducted in ASGM communities in Vietnam or Cambodia, and the one survey conducted in Thailand only included biomonitoring of human samples but no clinical examinations. The survey was conducted in the Phanom Pha gold mining area of Thailand, and it found that the environmental Hg contamination and open amalgam burning were the likely sources of the miners’ health problems, the Hg exposure at work surpassed permissible values, and the urine Hg levels indicated that the miners were being exposed to inorganic Hg [59]. Although no health assessments have been conducted in Cambodia, miners there have reported skin rashes, and animal deaths have been reported near the mining operations [43]. The atmospheric Hg level of these countries are also mentioned in Table 1 in terms of the level of Hg contamination. The gold-production region in central Sulawesi, Indonesia has the highest average 24-h ambient Hg values at 9172 ng/m^3^, which was nine times the WHO’s limit of 1000 ng/m^3^. The amalgam burning sites of both the Philippines and Myanmar also showed the high atmospheric Hg values at 314,000 ng/m^3^ and 74,000 ng/m^3^, respectively.

**Table 1:** Health assessment findings in SEA countries and Hg contamination levels.

Country	Year	Sample Size	Health Assessment Findings	References	Atmospheric Hg Level by Countries (ng/m^3^)
Indonesia (Galangan in Central Kalimantan and Talawaan in Northern Sulawesi)	2010	281	Ataxia, tremor, dysdiadochokinesia, etc. -Mean value of Hg-blood (μg/L); control group (A) (4.92), only living group in Kalimantan (B) (12.86) and Sulawesi (C) (7.05), panning workers in Kalimantan (D) (20.35) and Sulawesi (E) (15.18), smelting workers in Kalimantan (F) (38.92) and Sulawesi (G) (27.43). -Mean value of Hg-urine (μg/L); A (0.90), B (21.47), C (4.48), D (37.45), E (13.37), F (177.69), G (54.46). -Mean value of Hg-urine (μg/g creatinine); A (0.43), B (10.44), C (2.70), D (15.65), E (5.58), F (69.35), G (31.89). -Mean value of Hg-hair (μg/g); A (1.64), B (7.14), C (2.30), D (42.56), E (5.73), F (17.09), G (13.14)	Bose-O’Reilly, S. et al., 2010a [60]	9172 ± 16,422 (mean ± SD) [61]
Indonesia (Banten/Cisitu Village)	2015	28 children	Tremors in adults, and neurological deficits in children and teenagers such as developmental delays, hydrocephalus, deafness, vision disorders, and other congenital deformities	Ismawati, Y., 2015 [17]	
Indonesia (Banten/Cisitu Village)	2016	18	Typical signs and symptoms of chronic Hg intoxication (excessive salivation, sleep disturbances, tremors, ataxia, dysdiadochokinesia, pathological coordination tests, gray to bluish discoloration of the oral cavities, and proteinuria). The mean values of Hg-urine (μg/L) were increased in eight patients (>7 µg). All 18 people had increased hair levels (>1 µg Hg/g hair)	Bose-O’Reilly, S. et al., 2016 [12]	
Indonesia (Gorontalo)	2015	44	Bluish gums, Babinski reflex, labia reflex, tremor, rigidity, ataxia, alternating movements, and nystagmus in ASGM miners and inhabitants of Angrrek and Sumalata. -Hg-hair (μg/g); 14.2 μg	Arifin Y. I. et al., 2015 [62]	
Indonesia (Sulawesi, Makassar)	2016	40 gold workers and 17 residents as control	Tremors in the tongue, eyelid, finger, nose, pouring, posture holding, and Romberg test, unbalanced rigidity and ataxia, pathology reflex, sensory disturbance, constricted field of vision, and slow knee jerk and bicep reflexes. Hg-hair (μg/g); directly exposed group (10.8), indirectly exposed group (6.5), and control group (2.8)	Abbas H.H. et al., 2017 [63]	
Philippines (Mindanao/ Monkayo and Diwalwal) Davao as Control area	2000	323 (workers from Diwalwal, local families from Monkayo including children and a control group in Davao)	Fatigue, tremor, memory problems, restlessness, loss of weight, metallic taste, sleeping disturbances (reported symptoms), and intentional tremors, mainly fine tremors of the eyelids, lips, and fingers, ataxia, hyperreflexia, sensory disturbances, and bluish discoloration of the gums (symptoms) were observed in approximately 65% of the population in the Mt. Diwata area, and 85% in the ball mill and amalgam smelter workers. To a lesser but still not acceptable extent (approximately 33%) Hg intoxication (headache, vision problems, and nausea) was found in the nonoccupationally exposed population at Mt. Diwata and downstream in the Monkayo plain (38%). No Hg intoxication was found in the control area of Davao. -Hg-blood (μg/L); span < 0.25–107.6, median 8.2, arithmetic mean 11.48 -Hg-urine (μg/L); span < 0.25–294, median 2.5, arithmetic mean 11.08 -Hg-urine (μg/g creatinine); span < 0.1–196.3, median 2.4, arithmetic mean 8.40 -Hg-hair (μg/g); span 0.03–37.76, median 2.72, arithmetic mean 4. 14	Bose-O’Reilly, S. et al., 2000 [25]	314,000 [64]
Philippines (Apokon, Tagum, Davao del Norte)	2000	162 (school children aged 5–17 years)	Under-height, gingival discoloration, adenopathy, underweight and dermatologic abnormalities. -Total Hg-blood (μg/L); 0.757–56.88 -MeHg-blood (μg/L); 1.36–46.73 -Total Hg-hair (μg/g); 0.278–20.393 -MeHg-hair (μg/g); 1.36–46.73	Akagi, H. et al., 2000 [65]	
Myanmar (Mandalay Region/Chaung Gyi Village)	2020	29	Tremor, Ataxia, decreased lung function in miners. -Hg-hair (μg/g); miners 0.93 (0.72–1.44) (median–interquartile range) Nonminers 0.63 (0.53–0.67) (median–interquartile range)	Kyaw WT, 2020 [57]	74,000 [39]
Thailand (Phanom Pha)	2007	79 miners 59 school children	No clinical assessments were included. Hg-urine level; miners (μg/g creatinine) (22.85 ± 0.04 μg/g), School children (μg/g creatinine) (13.93 ± 0.33). Hg-hair level; miners (1.17 ± 0.05 μg/g), School children (0.93 ± 0.01)	UMBANGTALAD S, 2007 [59]	
Laos PDR -District of Chomphet (Houay Gno Village and Houay Koh), -District of Pak Ou (Latthahai Village and Pak Ou Village)	2004	191	The study observed neurological abnormalities in 56% of men (47 out of 83) and 41% of women (44 out of 107); however, only 16 % of men and 71% of women were using Hg. The author suggested considering other environmental and genetic factors as possible causes of the neurological abnormalities. Maximum level; Hg-blood level μg/L (12.2), Hg-urine level μg/L (15), and Hg-hair level μg/g (18.6)	Bose-O’Reilly, S. et al., 2004 [66]	

## 5. ASGM Problems in SEA Countries and a Sustainable Solution

As discussed above, ASGM community health assessments have generally been conducted by global projects and institutional researchers. The “Minamata Convention on Mercury” was adopted in 2013 at a diplomatic conference of the UNEP to resolve the problems resulting from anthropogenic Hg exposure. As of 2022, there were 128 signatories to the treaty out of 137 parties, including most SEA countries [67]. Projects such as the UNIDO global mercury project, which is focused on Hg environmental and health impact assessments in ASGM areas, are also being conducted. Nevertheless, despite these projects, the ASGM communities are still being exposed to these health hazards primarily because of poverty, a lack of information, and the ineffective monodisciplinary approach of academic researchers and governments. For example, researchers only conduct bottom-up ASGM community evaluations, whereas governments only focus on top-down ASGM rules and regulations, neither of which tackle the wicked nature of these issues. Furthermore, local medical professionals and health care service personnel are often unaware of chronic Hg intoxication symptoms or the other ASGM-related health issues, which results in misdiagnoses and a failure to provide prevention measures or early treatment.

Thus, the key to the sustainable resolution of these problems is to encourage transdisciplinary collaboration and active community participation to effectively share information, increase awareness, and add value to ASGM-related issues, which we refer to as transdisciplinary communities of practice (TDCoPs). The authors’ project, which is known as the Sustainable Regional Innovations for Reducing Environmental Pollution project, is a five-year project funded by the Institute for Humanity and Nature that runs from 2019 and 2023 to elucidate pathways to alleviate ASGM-related Hg contamination in Indonesia using a transdisciplinary approach. In collaboration with local stakeholders, several TDCoPs groups have been successfully formed in the study area to encourage sustainable community livelihoods and establish new industries to reduce the reliance on ASGM, promote environmental conservation, stop Hg vapor emissions into the atmosphere from amalgam burning, and promote community health [68]. Based on our experiences and following a review on the role of transdisciplinary research (TDR) and communities of practice (CoPs) in public health, TDCoPs are introduced in this article to demonstrate the environmental governance and health improvement possibilities for ASGM communities in SEA countries.

## 6. Environmental Governance and Community Health Improvements

### 6.1. General View of TDR and Its Role in Public Health

**Figure 1:**
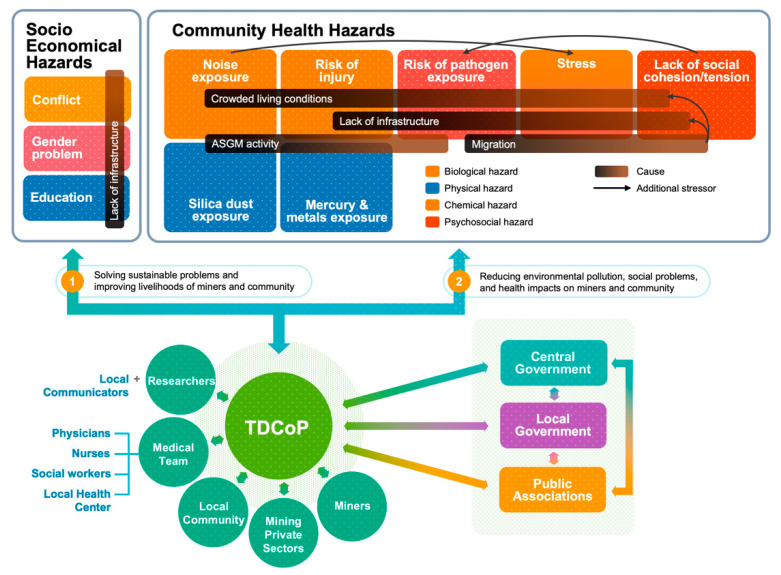
Transdisciplinary communities of practice (TDCoPs): a process to solve the wicked ASGM problems in SEA. Reproduced with permission from [69]. 2015, Basu N et al. The credit for the summary on the community health hazards goes to Basu N et al., 2015 “Integrated Assessment of Artisanal and Small-Scale Gold Mining in Ghana—Part 1: Human health Review”.

As the activities in the context of ASGM in SEA countries are similar to those in Ghana, Figure 1 summarizes the ASGM community health hazards in SEA countries based on previously published human health issues in Ghana [69] and proposes an environmental governance process to solve these hazards and improve community health. As summarized in Figure 1, the ASGM communities in SEA countries are affected by both socioeconomic hazards and community health hazards due to the nature of ASGM activities. These problems are defined as wicked problems due to the multifaceted, complicated, and inter-related nature of the challenges; thus, transdisciplinary (TD) rather than monodisciplinary approaches are needed that involve TDR for which interdisciplinary researchers work together with stakeholders to develop new knowledge to tackle the above-mentioned ASGM issues. Recent research has emphasized the value of interdisciplinary and transdisciplinary methods to advance scientific knowledge and solve important societal issues [70,71,72,73].

The term TDR initially appeared in the 1970s [74,75] and has various definitions. TDR is a research concept that involves a paradigm shift in research practice to solve complex societal challenges [76]. Although the TDR approach is similar to interdisciplinary and multidisciplinary approaches, it differs in the way the information is used and shared. In multidisciplinary approaches, everyone works on the same subject inside their own discipline’s limits, makes their own assumptions, and develops their own methodologies and frames of reference. In interdisciplinary approaches, the disciplinary boundaries are merged so that the assumptions, limitations, and ideologies are blended. Nevertheless, TDR emphasizes the integration of academic and nonacademic information to coproduce knowledge that transcends disciplinary and sectoral borders, which is achieved by increasing the participative cooperation between the various academic and experiential stakeholders, that is, the people with lay or “lived” knowledge [77,78,79]. For TDR activities to be effective, team members must be open to new ideas and must respect the views of other disciplines, and they must be willing to engage in the exchange of ideas, mutual debates, problem solving, and conflict resolution when there are opposing opinions or ideals [71,80]. A major advantage of TDR approaches is that they allow for the integration of concepts from multiple disciplines into a new, common, conceptual framework [81] and therefore can help establish new conceptual models that better elucidate the complex processes involved in producing and sustaining public health challenges and provide evidence for formulating solutions and public policy [80].

A recent review on the TD approach in public health stated that there had been transdisciplinary initiatives by scholars in the field of environmental health [82] and provided evidence that despite the obstacles and demands on researchers, the collaborations between social science and environmental health had the greatest potential to improve public and environmental health [83]. Another study introduced an approach that supported science-based dialog among stakeholders in cumulative burden assessments (CuBA) and conducted two workshops focused on this approach to examine its utility and feasibility. The results revealed that the stakeholders were able to conduct joint discourse on the cumulative burdens, learn about the technical and social issues related to CuBA, and coproduce knowledge. The study also suggested exploring ways the CuBA methodology could promote or hinder social learning and knowledge coproduction in diverse institutional, social, and political multiple environmental hazard situations [84].

Despite these efforts, however, TDR has several barriers of implementation between academic researchers and the nonacademic participants [85], the most challenging of which are encouraging collaboration between the community residents and the other nonacademic participants [86] and dealing with trust issues between the participants and the community. An evaluation of the members of a Canadian aging and technology research network was conducted to assess their perceptions and experiences with TDR, and its results found that TDR was effective in encouraging mutual learning and understanding and in attempting to address the complicated challenges in the targeted sector. Nevertheless, individual, systemic, and cultural impediments to the implementation of TDR were also found. The primary individual impediments were related to technology-based communication, the unfamiliar terminologies and goals, and the conflicting priorities when the business-oriented goals and the demands of the industrial partners did not line up with other priorities of academics and people from other fields. Many also found it hard to work with researchers who were more traditional and focused on a single discipline because this research approach tends to have a top-down decision-making structure, which does not work well with TDR collaborative team approach. Participants also said that a major systemic problem was that there was not enough time to meet the needs and interests of all stakeholders and to complete the research in a way that met the deadlines set by the funding agencies [77].

As TDR expects people from different backgrounds and interests to work together to solve problems and produce new ideas, there has been a significant interest in social theories on how people learn, such as CoPs, which could assist TDR participants and better facilitate their work [87].

### 6.2. CoPs in Public Health

Jean Lave and Etienne Wenger were the first to use the term CoPs in 1991 as part of their theory of situated learning to explain how knowledge and learning occurred in specific places, showing that the social relationships in these settings were more important for professional development than in the classroom. Within a group of CoPs, contextual learning occurs spontaneously as individuals become more proficient in the area of knowledge [88]. CoPs, which are defined as groups of people who learn together to resolve mutual concerns and make improvements through regular interactions, have three fundamental elements: domain, community, and practice [89]. In a CoPs learning community, there is a more equal relationship between the experts and the students; that is, the teachers and students work together. Although CoPs were initially formed to tackle specific problems using a team approach, this does not necessarily mean that CoPs can be artificially created [87]. Although CoPs can overcome barriers, foster new knowledge, and facilitate the coming together of normally segregated experts and individuals concerned about issues [89], participants remain within their formal organizations as they participate in CoPs. As they come and go from the CoPs, they learn about the local work practices.

CoPs have been used for TDR and have had great results. CoPs have been used in Canada to share and learn about climate change and health issues [90] and to develop professional and organizational public-health-development strategies in context. They have also been used as knowledge-to-action mobilizers for health practitioners in the Senior Health Research Transfer Network (later Seniors Health Knowledge Network (SHKN)) to improve the health of Ontario seniors [91]. However, although there have been significant benefits found from CoPs, some barriers have also been observed. A study that analyzed an SHKN case to examine the value of CoPs in facilitating system change found that the CoPs successfully served as an initiator to focus on best practice, research, and experience by providing a reflective learning cycle that motivated participants to work together; however, organizational and sector-level cultural norms that were driven by structural goals impeded the CoPs’ initiatives to modify behaviors in the long-term-care system [92]. Thus, it was concluded that unless provincial CoPs are properly supported in their attempts to transform systems, any improvements in elder health care would be lost. The authors also stated that the literature on CoPs lacked recommendations on how to utilize CoPs to encourage system change and suggested that more primary research on the CoPs’ functions and effects on system-level transformations was needed [92].

### 6.3. TDCoPs and Their Role in Securing Environmental Governance and Resolving ASGM Socioeconomic and Community Health Hazards in SEA Countries

#### 6.3.1. The Importance of TDCoPs

Matsumoto et al. (2022) claimed that there were three barriers faced by CoPs to overcome in TDR: “barriers of indifference”, where stakeholders abstain from participating due to disinterest in the problems; “barriers of position”, where stakeholders do not collaborate within the CoPs due to internal differences in position; and “barriers of continuity”, where the activities cease once researchers leave the CoPs [93]. Barriers of indifference occur when the stakeholder participation in CoPs is not enthusiastic; however, collaboration and knowledge development with stakeholders such as community inhabitants are critical when seeking to resolve environmental and sustainability issues [94]. Barriers of position occur when social class inequalities contribute to power imbalances [95] and negatively impact participation [86]. Although it is challenging to bring diverse stakeholders together because of their differing perspectives and attitudes [96,97], even if there is successful stakeholder collaboration, it is necessary to address the issue of sustainability, and it is here that the final barriers of continuity arise. Academic participants often leave when they have finished their research tasks [33]; thus, academic participants must commit themselves to being the CoPs’ coordinators in the early stages so that they can stabilize the CoPs before handing over the management to the local stakeholders [33]. If the academic scholars always lead the cooperation and knowledge generation and the stakeholders only “cooperate,” the activities would be more likely to cease when the researchers leave, leaving the challenges unaddressed [33].

These barriers to CoPs can be overcome when the CoPs take a transdisciplinary approach, as TDCoPs use transformative boundary objects (TBOs). Compared with CoPs, TDCoPs have more diversified stakeholder groups, which may also include “indifferent” participants, and they encourage boundary crossing and engagement between the members and the others around them. The stakeholder engagement then evolves into a collaboration, which then evolves into autonomy via learning and practice [33]. The TDCoP approach has been used to examine health, the environment, and socioeconomic and cultural aspects. Our ongoing TDCoPs experience of collaborating with ASGM workers, the community, and stakeholders has made the ASGM workers and their communities aware of the health impacts of their ASGM activities, which has made them more willing to explore and implement alternative sustainable livelihoods by adding value to their existing local and traditional knowledge, which they were made aware of through discussions with the stakeholders and researchers. One of the ongoing focuses of TDCoPs has been the Healthy and Resilient Village, which seeks to improve the socioeconomic condition and community health in the ASGM area in East Suwawa, Bone Bolango Regency in Gorontalo Province, Indonesia. The local government figures, such as heads of the villages, actively participate and collaborate with local researchers, local medical professionals, the local mining community, and academic researchers [68].

#### 6.3.2. The Process and Role of TDCoPs in Securing Environmental Governance and Resolving Socioeconomic and Community Health Hazards

The TDCoPs that will work towards resolving SEA’s ASGM issues could comprise (1) academic researchers from host countries and joint academic institutes with scientific knowledge who would be supported by local communicators; (2) medical teams, such as physicians, nurses, social workers, staff from local health centers, and specialists to deal with the long-term ASGM health problems; (3) local community members; (4) private mining sector members; (5) local miners to share their issues and livelihood goals; (6) public associations; and (7) authorities such as local governments and central governments (Figure 1). The transdisciplinary approach has been broadly applied to a range of long-term health care services in the community, with most published works being focused on rehabilitation [98,99], dementia care [100], the oral health care of stroke patients [100], palliative care in acute care settings [101], bladder health, and preventing lower urinary tract symptoms in women [102].

The starting point for the TDCoPs would be the local government, such as the village, district, or provincial departments concerned with ASGM-related environmental and health issues in the area, who would then initiate the development of a collaborative network of professionals to be responsible for taking practical actions. TDCoPs go through five stages: potential, coalescing, maturing, activity, and transformation [89]. During the potential stage, the participants share the purpose of the TDCoPs, collect the basic information from the diverse disciplines and build trust through dialog [93], which requires researchers to initially take the leading role and then gradually take a supporting role as the TDCoPs move into the transformation stage [93]. For instance, in the TDCoPs formed to deal with the ASGM issues, through dialog, (1) the miners and local community can discuss the health and socioeconomic problems they are facing and the traditional, cultural, and local knowledge unique to their living area; (2) the medical teams can share basic lay information on the general health of the community, the kind of support they can offer, the barriers they are facing, and knowledge on Hg-related health issues; (3) the private mining sectors can provide information on the issues between the ASGM miners and government officials, and especially those related to local laws, such as disputes over legislation and the permissions needed by government officials, miners, and mining companies; and (4) government officials can share knowledge about the official processes important to the ASGM profile. Several TDCoPs can be formed across multiple layers depending on the situation and the cultures, which are known as multilayer TDCoPs. The TDCoPs’ members determine the problems during this stage, and TBOs are identified, which leads to the formation of TDCoPs in the “coalescing” stage. Examples of TBOs are chronic Hg intoxication and respiratory health.

After the formation of TDCoPs, basic research is conducted. The local government take the roles of planning and action by collaborating with the upper and central governments; the local medical teams conduct surveys; and the researchers contribute research techniques, such as the methodologies for the environmental, health, and socioeconomic surveys in the study area. The local communicators and medical teams perform the surveys and work in collaboration with the community under the guidance of the local government. The key stakeholders oversee the surveys and research execution and support these activities by offering contact points for resident collaboration and information sharing. During the “maturing” and “activity” stages, the TDCoPs members achieve transformation by reflecting on the basic research findings and solving their own problems, at which time the researchers move into supportive roles by sharing their knowledge. Finally, the communities transform and make plans for practical activities that can evolve independently of the TDCoPs. The instigation of TDCoPs can reduce and/or solve sustainability problems and improve ASGM community livelihoods by reducing environmental pollution, resolving the social and health problems of both the miners and the mining community, and providing the community with an autonomous community-based team.

#### 6.3.3. A Brief Report of the Current TDCoPs Developed in Authors’ Project

Among several ongoing TDCoPs of the authors’ project, an example of a TDCoPs for securing environmental governance and resolving ASGM socioeconomic and health issues, called “Healthy and Resilient Village” (comprising several TDCoPs), by ASGM miners and owners, was formed in 2021 in East Suwawa, Bone Bolango Regency in Gorontalo Province, Indonesia by local and central stakeholders, the local medical team, and academic researchers after discussions since 2018. Healthy and Resilient Village is attempting to promote the public understanding of proper mining operations, the consequences of using Hg in ASGM, environmental issues, and community safety and health, and in the future, it will be transformed into an independent community that will tackle the sustainable environmental problems and improve the community livelihood [103].

## 7. Conclusions

This study presented a country-specific ASGM profile for SEA countries and outlined the community health hazards experienced because of the wicked problems in the areas of ASGM. Most monodisciplinary studies have focused on Hg-related health problem assessments, in which academic researchers have taken on the main roles. Although these studies were able to identify Hg-related issues in the ASGM communities, they have been unable to resolve the community health problems because of their monodisciplinary focus. Governments have also been trying to tackle the problems through the implementation of rules and regulations; however, the problems remain unsolved because there is no community involvement.

TD healthcare approaches have been found to have a positive effect on community health management in the long-run; moreover, the authors’ project in ASGM areas in terms of developing TDCoPs, which addresses ASGM-induced, complex, socioeconomical and health problems, have made significant progress in making the community sustainable. Therefore, based on these data, a TDCoPs in ASGM areas in SEA countries was proposed and would involve active participation by researchers, the medical profession, the local community, and other invested stakeholders to resolve the wicked ASGM problems and to provide environmental governance and community health improvement guidance in ASGM communities in SEA countries.

## Data Availability

Not applicable.
